# Catching Up with Popular Pesticides: More Human Health Studies Are Needed on Neonicotinoids

**DOI:** 10.1289/ehp.125-A41

**Published:** 2017-02-01

**Authors:** Nate Seltenrich

**Affiliations:** Nate Seltenrich covers science and the environment from Petaluma, CA. His work has appeared in *High Country News*, *Sierra*, *Yale Environment 360*, *Earth Island Journal*, and other regional and national publications.

Prior to 2000, neonicotinoid chemicals were virtually unknown, by farmers or anyone else. They have since become the most widely used class of agricultural insecticides on the planet.^1^ With their rise has come evidence they are contributing to devastating losses of honeybees,^2^ ^,^ ^3^ yet despite widespread human exposure through fruits and vegetables, however, little research has been conducted on potential effects on human health, according to a review in *EHP*.^4^

**Figure:**
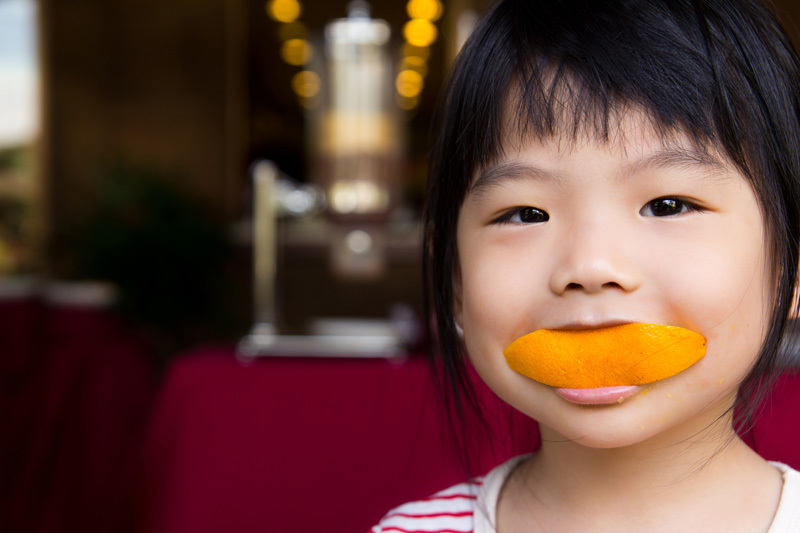
Neonicotinoids are important in protecting young orange trees from a deadly disease called citrus greening.^19^ Yet use of the insecticides on orange trees, in particular, may threaten honeybee colonies because orange blossoms are so attractive to pollinators.^20^ Impacts of neonicotinoids on humans, meanwhile, are poorly understood. © Kenishirotie/Shutterstock

“Over 15 years we went from nonuse to widespread use,” says senior author Melissa Perry, who is chairman of the Department of Environmental and Occupational Health at the Milken Institute School of Public Health. “That’s very, very rapid distribution in the marketplace. We simply don’t have the systems in toxicology and environmental exposure analysis to quickly shift to evaluate the impacts on human health.”

Perry and colleagues initiated their review by searching the peer-reviewed literature for epidemiological studies published between 2005 and 2015 that addressed human health effects of neonicotinoids. What they found was surprising, Perry says: A total of just eight studies met their parameters. Half of those addressed acute exposures, including accidental or intentional self-poisoning,^5^ ^,^ ^6^ ^,^ ^7^ ^,^ ^8^ and half addressed chronic environmental exposures.^9^ ^,^ ^10^ ^,^ ^11^ ^,^ ^12^

None of the environmental exposure studies prospectively assessed neonicotinoid-treated produce as the primary exposure pathway. Instead, two considered exposures through air and water based on proximity to agricultural fields, and one examined exposures through the use of flea and tick medications on pets. The fourth did not isolate a single pathway but assessed exposure by measuring urinary levels of *N*-desmethyl-acetamiprid (DMAP), a metabolite of the common neonicotinoid acetamiprid.

The findings of the various studies ran the gamut. A small study of people who planted treated seedlings found no associations between occupational exposure and adverse health effects,^5^ while another reported 2 fatalities out of 57 cases of neonicotinoid ingestion.^8^ The chronic studies reported associations between neonicotinoid exposures and at least some of the outcomes evaluated in each study, including congenital heart defects,^9^ anencephaly,^12^ and autism spectrum disorders.^10^ Another chronic study found that exposure was more likely to have occurred in people with a specific set of symptoms, including memory loss and finger tremor.^11^

The authors also assessed the internal validity, or risk of bias, in the studies they reviewed. “Bias” in this sense refers not to intentional influence, but to any quality of the study that may unintentionally steer findings in one direction or another. They evaluated risk of bias in nine areas related to study design and reporting and found methodological shortcomings across the eight studies, representing an overall “probably high risk of bias” and warranting “low to moderate confidence” in the findings. All eight were nonetheless retained in order to facilitate the review.^4^

Estimates on usage vary, but it is generally believed that most corn, at least one-third of soybeans, and a wide variety of other cereal crops, oil crops, fruits, and vegetables are treated with neonicotinoids, primarily through seed coatings and soil injections.^13^ These chemicals are systemic, meaning they are absorbed by the plant, mostly through the roots, and then circulate throughout its tissues, killing susceptible insects that feed on them. Residues cannot be washed off and are directly ingested by consumers.^14^

Review coauthor Abee Boyles, a health scientist administrator with the National Institute of Environmental Health Sciences, assisted in the development of the risk-of-bias assessment method and its implementation for this study. In late 2015 she also helped launch an effort at the National Toxicology Program, spawned by her collaboration with Perry, to examine in an even broader sense the state of the science on human health impacts of neonicotinoids, including *in vitro* and animal studies excluded from the present review. A report is due out later this year.

“The target that neonicotinoids are designed to hit is very insect-specific. But what we don’t know is if it could potentially hit something else in humans, maybe on a lower level,” Boyles says. “So I think the question is, what is the evidence available on potential other effects that we just haven’t looked for yet?”

Neurological effects in humans are a logical focus for new research, given how neonicotinoids act on insects and how nicotine, a relative of these insecticides, affects humans, says pesticide expert and former Washington State University professor Charles Benbrook. He also points out the need to assess the possible synergistic effects of prenatal exposures to neonicotinoids in combination with other herbicides and fungicides, saying, “Virtually all Americans, on a daily basis, incur such [mixed] exposures.”

The U.S. Environmental Protection Agency currently considers neonicotinoids to be of relatively low risk to mammals, Benbrook says. However, while the studies required for pesticide registration showed these compounds to be less toxic to mammals than to insects, toxic effects were nevertheless noted in animal studies.^15^ More recent laboratory and ecological field studies indicate that neonicotinoids can have adverse effects on mammals at sublethal doses,^15^ ^,^ ^16^ ^,^ ^17^ and some neonicotinoid metabolites may be as or more toxic, compared with the parent compound.^18^

“Revisiting the human health effects of neonicotinoids is really critical now, especially with a focus on low-dose endocrine effects,” Benbrook says, “because if in fact the current risk assessments are based on unrealistically high chronic reference doses, it will throw into doubt the registrations of many of the insecticides that now bear most of the brunt in managing key fruits and vegetables.”
